# Supplementary material for: Complexome profiling of the Chlamydomonas psb28 mutant reveals TEF5 as an early PSII assembly factor
Source: Plant Cell. 2025 Mar 17;37(6):koaf055. doi: 10.1093/plcell/koaf055 (PMC12164590; doi:10.1093/plcell/koaf055)
Supplement: koaf055_Supplementary_Data [file koaf055_supplementary_data.zip › psb28_interactive.zip!/plots/intensity/qualityPlots/peptides/_Cre01.g027550.t1.1.html]

### Cre01.g027550.t1.1

Trivial name:   
SeqCoverage [median]: 43.60  
Mass: 41.74 kDa  
  
 

| Sequence | MissedCleavage | Mass [Da] | wtIntensity | muIntensity | Score |
| --- | --- | --- | --- | --- | --- |
| ALAEDTLPAGLR | 0 | 1225.67 | 12093.79 | 38793.03 | 176.33 |
| ALAEDTLPAGLRGDPAGYPIGK | 1 | 2181.14 | 11343.16 | 36100.50 | 95.09 |
| AVYGVLK | 0 | 748.45 | 0.00 | 6678.93 | 97.64 |
| AVYGVLKEAEHR | 1 | 1370.73 | 483.81 | 0.00 | 37.68 |
| DPDTFRPER | 1 | 1131.53 | 1634.62 | 6236.78 | 57.59 |
| FFEPNSNPDFGGK | 0 | 1454.65 | 13411.87 | 51098.60 | 169.52 |
| FLVDMRGEEPTNK | 1 | 1534.74 | 23398.69 | 45769.88 | 91.86 |
| GDPAGYPIGK | 0 | 973.49 | 7449.62 | 12569.85 | 99.44 |
| LAVPAEK | 0 | 726.43 | 22735.59 | 45280.99 | 111.73 |
| MYPQPPILIR | 0 | 1226.68 | 8784.57 | 45459.88 | 147.34 |
| RALAEDTLPAGLR | 1 | 1381.77 | 1073.64 | 3782.12 | 62.89 |
| RALAEDTLPAGLRGDPAGYPIGK | 2 | 2337.24 | 0.00 | 4259.19 | 40.01 |
| RTPSGGSGSGAPGAAAK | 1 | 1427.71 | 9477.14 | 14731.49 | 106.38 |
| SPYLWKDPDTFRPER | 2 | 1905.94 | 4515.74 | 19779.57 | 65.09 |
| TPSGGSGSGAPGAAAK | 0 | 1271.61 | 3992.04 | 7243.13 | 142.10 |
| TPTLEDLK | 0 | 915.49 | 0.00 | 6891.14 | 113.71 |
| VAEDAEALQNR | 0 | 1214.59 | 17070.07 | 35440.66 | 223.84 |
| VAEDAEALQNRDYSK | 1 | 1707.81 | 12553.31 | 24757.72 | 135.14 |
| VGMATGATIHTANGLSMR | 0 | 1786.88 | 0.00 | 2728.51 | 74.43 |
| VLEEVDR | 0 | 858.44 | 2427.16 | 2483.48 | 89.55 |
| VSDPSLLR | 0 | 885.49 | 29928.03 | 20306.42 | 138.55 |
|  |  |  | 182372.9 | 430391.9 |  |
